# The safety and efficacy of nocturnal noninvasive positive pressure ventilation in patients with chronic obstructive pulmonary disease: a systematic review and meta-analysis

**DOI:** 10.3389/fmed.2026.1882152

**Published:** 2026-07-14

**Authors:** Ganying Huang, Yitao Zhou, Yangbin Zhou, Huijie Yang, Jinhong Wu, Weihong Zhu

**Affiliations:** 1 Department of Emergency, Affiliated Hangzhou First People’s Hospital, School of Medicine, Westlake University, Hangzhou, Zhejiang, China; 2 School of Nursing, Zhejiang Chinese Medical University, Hangzhou, Zhejiang, China

**Keywords:** blood gas analysis, chronic obstructive pulmonary disease (COPD), exercise capacity, nocturnal NIPPV, systematic review

## Abstract

**Background:**

The efficacy of nocturnal noninvasive positive pressure ventilation (NIPPV) in stable COPD remains controversial. This study evaluates the safety and clinical impact of nocturnal NIPPV compared with conventional therapy.

**Methods:**

A systematic search of PubMed, Embase, Cochrane Library, Web of Science, CNKI, and Wanfang was conducted from inception to July 26, 2025. Eligible randomized controlled trials (RCTs) published from 2015 onward and evaluating nocturnal NIPPV in stable COPD were included. Primary outcomes were arterial blood gases (PaCO2, PaO2, pH); secondary outcomes included pulmonary function, 6-min walking distance (6MWD), and health-related quality of life (HRQoL). Pooled effects were calculated using fixed- or random-effects models. PROSPERO Registration (CRD42024608837).

**Results:**

Eight RCTs (736 patients) were analyzed. Nocturnal NIPPV significantly reduced PaCO2 and improved PaO2, with no significant change in pH. Regarding pulmonary function, FVC showed a non-significant trend toward improvement, and no significant differences were found for FEV1 or FEV1/FVC. Notably, 6MWD showed significant improvement in the NIPPV group. For HRQoL, significant gains were observed in the Severe Respiratory Insufficiency (SRI) questionnaire, and COPD Assessment Test (CAT) scores were significantly reduced. No serious adverse events were reported.

**Conclusion:**

Nocturnal NIPPV is a safe intervention for stable COPD, offering physiological and functional benefits by improving gas exchange and exercise capacity, while pulmonary-function effects were less consistent. However, its effects on spirometric airflow limitation and patient-reported outcomes should be interpreted cautiously because outcome instruments and the number of available studies differed. Further high-quality RCTs with standardized protocols are needed to define its optimal role in long-term management.

## Introduction

Chronic obstructive pulmonary disease (COPD) is a progressive respiratory disorder characterized by persistent airflow limitation and chronic respiratory symptoms, leading to substantial morbidity, impaired quality of life, and increased mortality worldwide (1). In patients with advanced disease, chronic hypercapnic respiratory failure is a frequent and clinically important complication, reflecting ventilatory muscle overload, impaired gas exchange, and altered respiratory mechanics (2). Identifying effective long-term management strategies for stable hypercapnic COPD therefore remains a major clinical challenge.

Non-invasive positive pressure ventilation (NIPPV) is well established as the standard of care for acute hypercapnic respiratory failure in patients with COPD (3, 4). Beyond the acute setting, long-term NIPPV has been proposed as a therapeutic option for patients with stable COPD, with the aim of improving gas exchange, unloading respiratory muscles, and enhancing ventilatory efficiency. Several studies and meta-analyses have reported that long-term NIPPV may reduce arterial carbon dioxide tension (PaCO₂) and increase arterial oxygen tension (PaO₂), particularly when higher inspiratory pressures are applied (5–7). However, its effects on lung function, exercise capacity, and patient-reported outcomes have been inconsistent.

The role of NIPPV in stable COPD remains controversial. While some investigations have demonstrated improvements in blood gas parameters, functional capacity, and quality of life, others have failed to show consistent benefits in airflow limitation, symptom burden, or long-term outcomes. These discrepancies may be attributed to substantial heterogeneity across studies, including differences in patient selection, ventilatory strategies, treatment duration, and outcome definitions (8, 9). Importantly, many previous studies and meta-analyses have combined daytime and nocturnal NIPPV use, which may obscure the specific effects of nocturnal ventilation (10).

Nocturnal NIPPV represents a clinically relevant intervention, as nocturnal hypoventilation and sleep-related respiratory disturbances may contribute to sustained daytime hypercapnia in patients with stable COPD (11). Nevertheless, the isolated effects of nocturnal NIPPV on arterial blood gas parameters, pulmonary function, exercise capacity, and symptom-related outcomes have not been comprehensively and consistently evaluated.

Therefore, we conducted a systematic review and meta-analysis to compare nocturnal NIPPV with standard conventional therapy in patients with stable COPD. This study aimed to provide an updated and focused synthesis of evidence regarding the effects of nocturnal NIPPV on blood gas parameters, pulmonary function, exercise capacity, and patient-reported outcomes, thereby clarifying its clinical role in the long-term management of stable COPD.

## Methods

### Protocol and registration

This meta-analysis was conducted in accordance with the Preferred Reporting Items for Systematic Reviews and Meta-Analyses (PRISMA) statement. The study protocol was registered in PROSPERO (Registration No. CRD42024608837). Two amendments to the registered PROSPERO protocol were implemented during the review process. First, the database search, originally conducted on August 7, 2024, was updated on July 26, 2025, using identical search strategies across all six databases to capture newly published studies; Second, during eligibility screening, a restriction was applied to include only RCTs published from 2015 onward, to focus on contemporary nocturnal NIPPV practice reflecting current high-intensity ventilation strategies and guideline recommendations (see Limitations for further discussion). Both amendments have been documented in an updated PROSPERO record.

### Search strategy

A comprehensive literature search was performed in the Cochrane Library, Embase, PubMed, Web of Science, China National Knowledge Infrastructure (CNKI), and Wanfang databases to identify relevant studies published up to July 26, 2025. The complete search strategy is provided in Supplementary Table 1; for reproducibility, the Web of Science query was: TS = ((“noninvasive ventilation” OR “non-invasive ventilation” OR NIPPV OR NIV) AND (COPD OR “chronic obstructive pulmonary disease” OR “chronic obstructive lung disease”) AND (nocturnal OR night* OR sleep*) AND (random* OR trial OR placebo)). The PubMed search strategy combined the Medical Subject Headings (MeSH) terms “Noninvasive Ventilation,” “Pulmonary Disease, Chronic Obstructive,” and “Nocturnal.” In addition, studies involving patients with chronic obstructive pulmonary disease (COPD) complicated by coexisting sleep disorders were excluded to reduce clinical heterogeneity. Reference lists of eligible studies and relevant reviews were manually screened to identify additional potentially eligible articles. Duplicate records were removed using EndNote software.

Trial registries and grey-literature sources were not searched. To minimize publication bias within the review scope, we searched six databases without language restriction and manually screened reference lists; formal funnel plot or Egger testing was not performed because fewer than 10 studies were included.

### Selection criteria

The inclusion criteria were: (1) Compared conventional standard therapy combined with noninvasive positive pressure ventilation (NIPPV) with conventional standard therapy alone; (2) Reported at least one primary outcome related to arterial blood gas parameters, including partial pressure of carbon dioxide (PaCO₂), partial pressure of oxygen (PaO₂), or pH; (3) Reported at least one secondary outcome, including lung function indices (forced expiratory volume in 1 s [FEV₁], forced vital capacity [FVC]), mortality, or 6-min walking distance (6MWD); (4) No restrictions on language.

The exclusion criteria were: (1) Did not involve NIPPV as an intervention; (2) Included patients with COPD complicated by sleep-related breathing disorders; (3) Were published before 2015, consistent with the amended PROSPERO eligibility criterion. This restriction was adopted to focus the synthesis on trials conducted under the contemporary high intensity, PaCO₂ targeted nocturnal NIPPV paradigm; the two pre-2015 RCTs excluded solely on this basis are detailed in Supplementary Table 2; (4) Were meta-analyses, systematic reviews, case reports, editorials, animal studies, letters, comments, conference abstracts, or ongoing studies.

### Data extraction and quality assessment

Two investigators (Zhou and Zhou) independently screened titles, abstracts, and full texts and extracted relevant data. Any disagreements were resolved through discussion with a third investigator (Huang). Extracted data included author information, publication year, study design, sample size, patient characteristics, intervention details (including IPAP, EPAP, back-up rate, and ventilation targets when available), patient characteristics (including chronic hypercapnia status, long-term oxygen therapy, exacerbation history, and GOLD stage when available), mortality, and outcome measures.

The methodological quality of randomized controlled trials (RCTs) was assessed using the Cochrane Risk of Bias 2.0 Tool (12).

### Statistical analysis

Statistical analyses were conducted using Review Manager software (RevMan, version 5.4; The Cochrane Collaboration). Continuous outcomes were expressed as mean differences (MDs) or standardized mean differences (SMDs), both with 95% confidence intervals (CIs). A two-sided *p*-value < 0.05 was considered statistically significant. Statistical heterogeneity among studies was assessed using the *I*^2^ statistic. An *I*^2^ value ≤ 50% indicated low heterogeneity, and a fixed-effects model was applied; an *I*^2^ value > 50% indicated substantial heterogeneity, and a random-effects model was used. Due to the limited number of included studies (*n* < 10), a formal assessment of publication bias using funnel plots or Egger’s test was not performed, in accordance with the Cochrane Handbook recommendations.

Leave-one-out sensitivity analyses and subgroup analyses were planned in the protocol; however, they were not performed when fewer than three studies or insufficiently comparable data were available for an outcome.

## Results

### Study selection

A total of 848 records were identified through database searching, including PubMed (*n* = 503), Cochrane Library (*n* = 83), Embase (*n* = 65), Web of Science (*n* = 70), CNKI (*n* = 50), and Wanfang (*n* = 77). No additional records were identified through other sources. After removing duplicate records, 697 studies remained for title and abstract screening.

Of these, 644 records were excluded because they were not relevant to the research topic (*n* = 542) or did not meet the required study design (*n* = 102). Fifty-three full-text articles were subsequently assessed for eligibility. Among them, 45 articles were excluded due to ongoing study status (*n* = 6), irrelevant outcomes (*n* = 19), inappropriate study type (*n* = 12), pre-2015 publication after otherwise potentially relevant screening (*n* = 2), or inclusion of patients with coexisting sleep disorders (*n* = 6).

**Figure 1:**
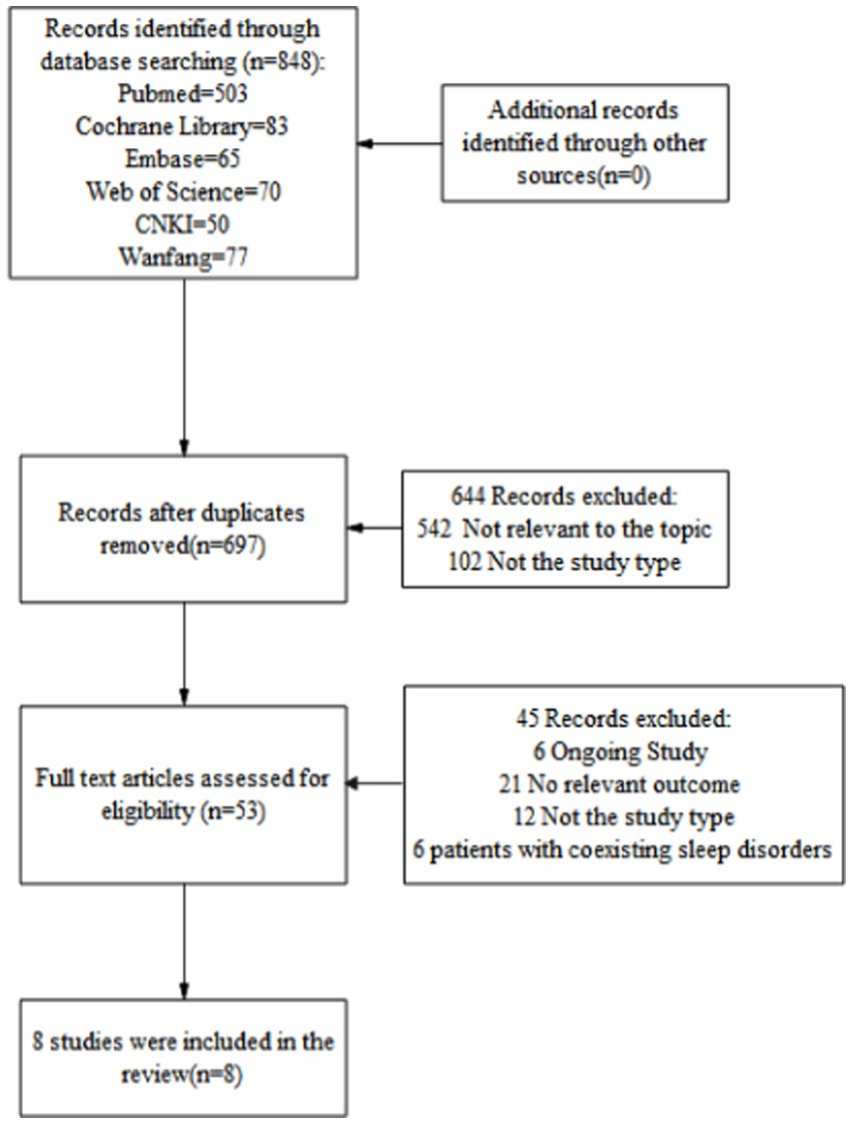
Flow diagram: the study selection procedure. The diagram illustrates the identification, screening, eligibility, and inclusion of randomized controlled trials comparing nocturnal NIPPV with conventional therapy.

Ultimately, 8 studies (9, 13–19) met the inclusion criteria and were included in the meta-analysis, comprising a total of 736 patients with COPD. The study selection process is illustrated in Figure 1, and the main characteristics of the included studies are summarized in Table 1.

**Table 1:** Baseline characteristics of the included studies.

Reference	Country	Sample size(I/C)	Duration	PH		PaCO2 (mmHg)		PaO2 (mmHg)		Lung function FEV1(L / %)	
				Intervention	Control	Intervention	Control	Intervention	Control	Intervention	Control
Murphy et al. (21)	UK	57/59	12 months	NR	NR	47.3 ± 6.8	51.8 ± 7.1	63.8 ± 8.3	62.1 ± 9.0	0.82 ± 0.35	0.80 ± 0.33
Duiverman et al. (13)	Netherlands	33/34	6 months	NR	NR	54.0 ± 4.5	57.8 ± 5.2	60.0 ± 9.0	51.0 ± 9.8	0.90 ± 0.30/35.0 ± 11	0.80 ± 0.30/34.0 ± 10
Xie et al. (9)	China	132/137	6 months	NR	NR	48.2 ± 4.5	53.1 ± 5.1	84.3 ± 6.1	76.1 ± 5.9	38.4 ± 8.5	37.9 ± 8.2%
Zhou et al. (14)	China	22/22	8 weeks	7.39 ± 0.04	7.38 ± 0.05	49.5 ± 5.2	52.1 ± 5.5	78.2 ± 6.5	75.1 ± 6.1	0.78 ± 0.22	0.76 ± 0.20
Xu (16)	China	29/29	Follow-up	7.35 ± 0.08	7.36 ± 0.09	42.1 ± 4.0	50.3 ± 6.1	98.7 ± 7.7	85.1 ± 7.3	NR	NR
Zhou et al. (15)	China	34/34	6 months	NR	NR	42.5 ± 4.8	46.2 ± 5.1	88.4 ± 7.2	82.5 ± 6.8	NR	NR
Ge et al. (18)	China	14/28	8 weeks	NR	NR	NR	NR	NR	NR	1.02 ± 0.28	0.86 ± 0.24
Cheng and Peng (17)	China	37/35	12 months	7. 37 ± 0. 09	7. 30 ± 0. 07	42.04 ± 4.13	47.94 ± 6.89	98.59 ± 7.81	78.78 ± 6.50	68.45 ± 8.59	43.79 ± 8.54

Reference	Country	Sample size (I/C)	Lung function FVC(L)		Lung function FEV1/FVC(%)		6MWD (meters)		CAT/SRI (points)	
			Intervention	Control	Intervention	Control	Intervention	Control	Intervention	Control
Murphy et al. (19)	UK	57/59	NR	NR	NR	NR	NR	NR	SRI: 52.1 ± 16	SRI: 45.8 ± 15
Duiverman et al. (13)	Netherlands	33/34	NR	NR	NR	NR	231 ± 90	179 ± 63	SRI: 61.0 ± 12	SRI: 50.0 ± 11
Xie et al. (9)	China	132/137	NR	NR	36.12 ± 6.45	35.88 ± 6.32	345 ± 42	308 ± 38	CAT: 15.2 ± 3.1	CAT: 18.5 ± 3.5;
Zhou et al. (14)	China	22/22	NR	NR	38.5 ± 8.4	37.9 ± 8.1	325 ± 45	298 ± 40	SRI 62.5 ± 10.2	SRI:55.4 ± 9.7
Xu (16)	China	29/29	1.74 ± 0.42	1.76 ± 0.38	48 ± 11%	0.47 ± 9%	NR	NR	NR	NR
Zhou et al. (15)	China	34/34	1.08 ± 0.25	0.95 ± 0.21	52.4 ± 6.8	48.5 ± 5.6	365.2 ± 41.5	332.6 ± 38.4	CAT 14.2 ± 3.5	CAT:17.8 ± 3.2
Ge et al. (18)	China	14/28	NR	NR	52.4 ± 6.8	44.5 ± 5.6	386.4 ± 42.1	324.5 ± 38.6	CAT: 18.4 ± 3.6	CAT: 23.5 ± 4.2
Cheng and Peng (17)	China	37/35	1.72 ± 0.36	1.38 ± 0.40	NR	NR	198.84 ± 34.68	167.89 ± 38.79	NR	NR

NR, not reported. Long-term oxygen therapy use, exacerbation history, GOLD stage, ventilatory settings (IPAP/EPAP/back-up rate), and ventilation targets were not consistently reported across the included studies and therefore could not be tabulated comparably or used for subgroup meta-analysis.

Six of the eight included studies were conducted in China, with the remaining studies from the United Kingdom and the Netherlands. Reporting of long-term oxygen therapy, exacerbation history, GOLD stage, IPAP/EPAP, back-up rate, and ventilation targets varied across trials and was insufficient for subgroup meta-analysis.

Regarding baseline ventilatory status, most included studies enrolled stable COPD patients with chronic hypercapnia or hypercapnic respiratory failure; where reported, aggregate baseline PaCO2 values ranged from 42.04 to 57.8 mmHg. No included trial was designed to enroll a normocapnic stable COPD population, although individual-level normocapnic status could not be fully verified from aggregate data.

### Risk of bias

**Figure 2:**
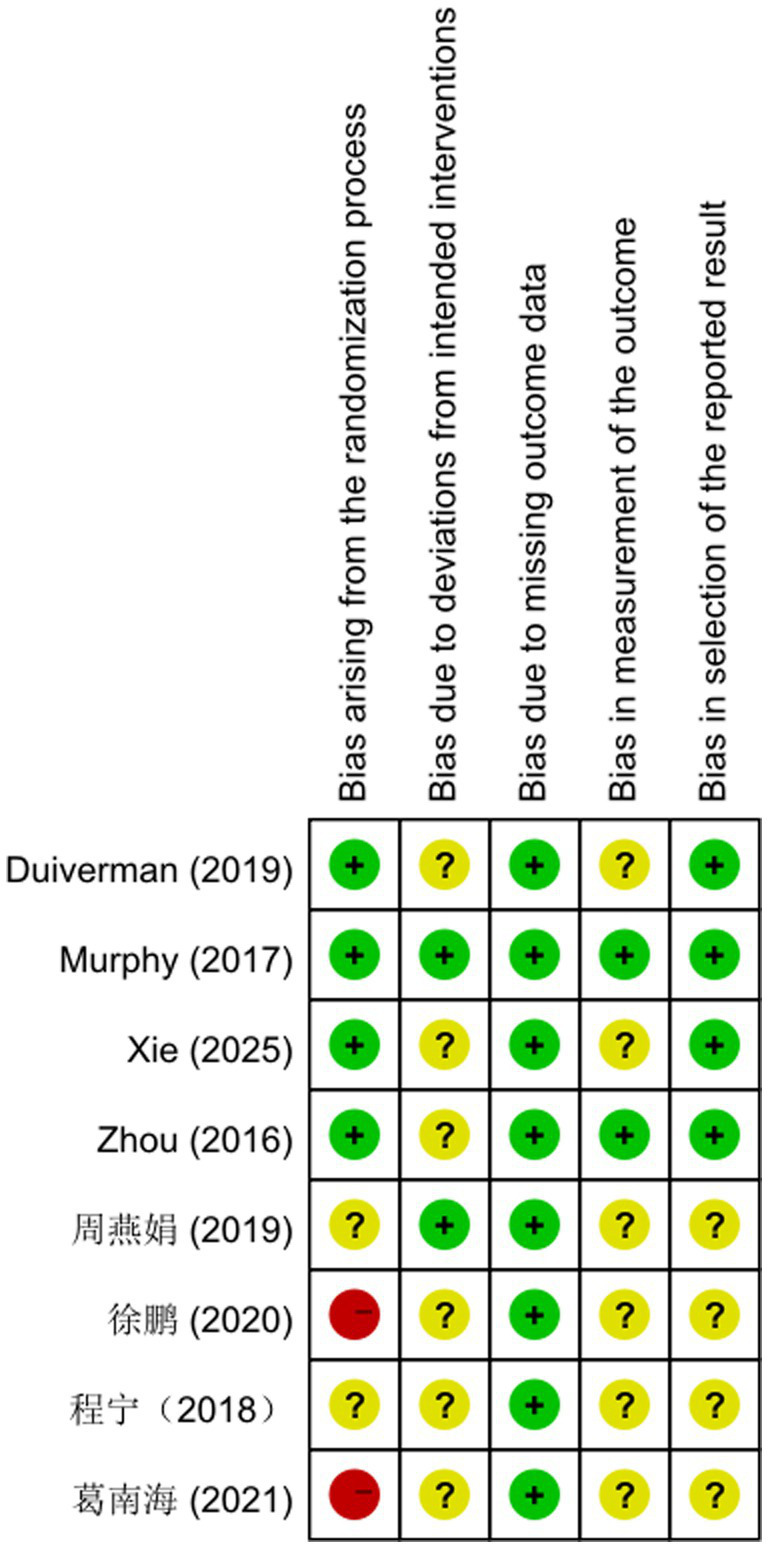
Risk of bias summary. Review of authors’ judgments about each risk of bias domain for each included study using the Cochrane Risk of Bias 2.0 tool.

**Figure 3:**
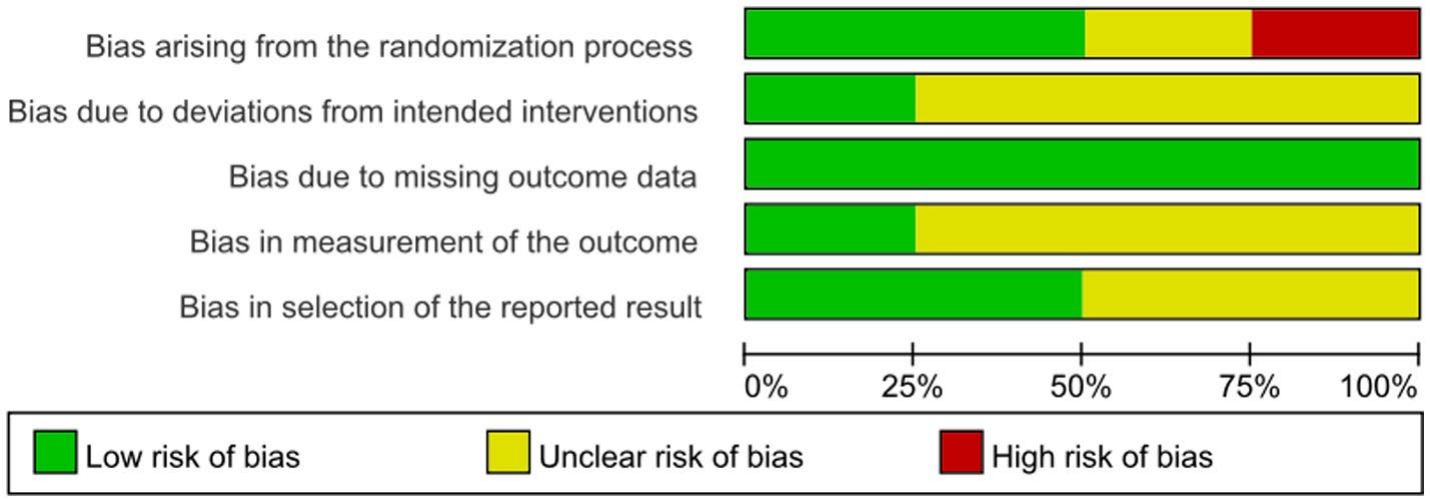
Risk of bias graph. Review of authors’ judgments about each risk of bias domain presented as percentages across all included studies.

The risk of bias assessment for the included randomized controlled trials is presented in Figures 2, 3. Overall, most domains were judged as having a “low risk of bias” or “some concerns” according to the Cochrane Risk of Bias 2.0 tool. Due to the limited number of included studies (*n* < 10), publication bias was not formally assessed using funnel plots, as the statistical power of such tests is insufficient to provide reliable interpretations under these conditions, as per the Cochrane Handbook recommendations. However, a comprehensive search across multiple databases was performed to minimize the risk of missing relevant literature.

### The outcomes

#### Blood gas analysis

**Figure 4:**
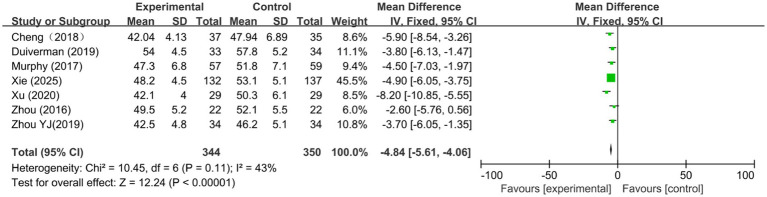
Forest plot PCO2(mmHg). This forest plot evaluates the impact of nocturnal NIPPV on arterial carbon dioxide tension (PCO2). A fixed-effects model was applied as low statistical heterogeneity was observed among the seven included studies (I2 = 43%, *p* = 0.11). The pooled analysis showed a statistically significant reduction in PCO2 levels in the nocturnal NIPPV group compared with the conventional therapy group (MD = −4.84, 95% CI: −5.61 to −4.06; *p* < 0.00001).

For blood gas parameters, the pooled analysis showed a statistically significant reduction in PaCO₂ in the intervention group compared with the control group (Figure 4). A statistically significant improvement in PaO₂ was also observed in the intervention group (Supplementary Figure 1). In contrast, no statistically significant difference was found in arterial pH between the intervention and control groups (Supplementary Figure 2). All analyses were performed using a random-effects model, and substantial heterogeneity was observed across studies.

#### Pulmonary function

For pulmonary function outcomes, the pooled analysis of forced vital capacity (FVC) showed a non-significant trend toward improvement in the intervention group (MD = 0.15, 95% CI 0.02 to 0.33; *p* = 0.09). A random-effects model was applied because substantial heterogeneity was observed among the included studies (I^2^ = 72%, *p* = 0.03) (Supplementary Figure 3).

However, no statistically significant differences were observed between groups for FEV₁ measured in liters (Supplementary Figure 4), FEV₁ expressed as percentage of predicted values (Supplementary Figure 5), or the FEV₁/FVC ratio (Supplementary Figure 6).

#### Symptoms and functional outcomes

**Figure 5:**
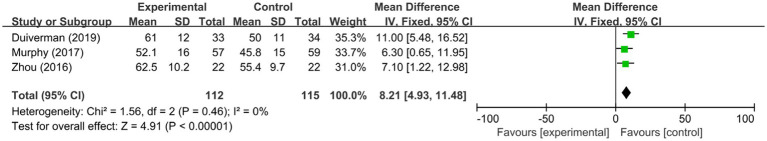
Forest plot SRI (points). This forest plot evaluates the impact of nocturnal NIPPV on health-related quality of life using the SRI questionnaire. A fixed-effects model was applied as no statistical heterogeneity was observed among the three included studies (I2 = 0%, *p* = 0.46). The pooled analysis demonstrated a statistically significant improvement in quality-of-life scores for the nocturnal NIPPV group compared with the conventional therapy group.

Regarding symptom-related outcomes, the pooled analysis of the St. George’s Respiratory Questionnaire–related index (SRI) showed a statistically significant improvement in the intervention group compared with the control group, with no heterogeneity observed among studies (MD = 8.21, 95% CI 4.93–11.48; *I*^2^ = 0%, *p* = 0.46) (Figure 5).

The pooled analysis of COPD Assessment Test (CAT) scores demonstrated a statistically significant reduction in the nocturnal NIPPV group compared with the control group (MD = −3.49, 95% CI 4.17 to −2.81; *p* < 0.00001) (Supplementary Figure 7).

**Figure 6:**
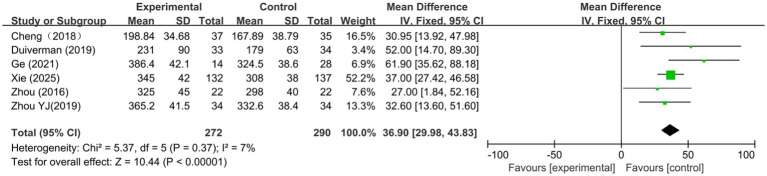
Forest plot 6MWD (meters). Description: This forest plot evaluates the impact of nocturnal NIPPV on functional exercise capacity, measured by the 6-min walking distance (6MWD) in meters. A fixed-effects model was applied, as low statistical heterogeneity was observed among the six included studies (I2 = 7%, *p* = 0.37). The pooled analysis demonstrated a statistically significant improvement in exercise capacity in the nocturnal NIPPV group compared with the conventional therapy group (MD = 36.90, 95% CI: 29.98 to 43.83; *p* < 0.00001; MD = 8.21, 95% CI: 4.93 to 11.48; *p* < 0.00001).

For exercise capacity, the pooled analysis of the 6-min walking distance (6MWD) demonstrated a statistically significant improvement in the intervention group compared with the control group (MD = 36.90, 95% CI 29.98–43.83), with low heterogeneity among studies (*I*^2^ = 7%, *p* = 0.37) (Figure 6).

Mortality was planned as a secondary outcome; however, mortality endpoints were not consistently reported using comparable definitions and follow-up periods across the included RCTs. Therefore, a pooled mortality analysis was not performed.

## Discussion

This meta-analysis evaluated the effects of nocturnal non-invasive positive pressure ventilation (NIPPV) in patients with stable chronic obstructive pulmonary disease (COPD). The main findings indicate that nocturnal NIPPV is associated with significant improvements in arterial blood gas parameters, particularly reductions in PaCO2 and increases in PaO2, whereas no statistically significant effect was observed on arterial pH. These findings support the role of nocturnal NIPPV in correcting chronic hypercapnia and improving oxygenation in stable COPD. The significant reduction in PaCO2 observed in our analysis aligns with the evolving paradigm of ‘high-intensity’ NIPPV, which prioritizes the normalization of blood gas levels through higher inspiratory pressures. Recent perspectives, including the GOLD 2024 Report (20), emphasize that long-term NIPPV is most effective in stable COPD when titration aims for a substantial decrease in hypercapnia, a strategy that has been shown to reduce mortality and rehospitalization risk in persistent hypercapnic patients (19, 21). Beyond blood gas outcomes, FVC showed only a non-significant trend toward improvement, while no significant changes were observed in airflow limitation-related parameters, including FEV1 (expressed either in liters or as percentage of predicted values) and the FEV1/FVC ratio. This pattern does not support a consistent spirometric improvement in fixed airflow limitation; any possible effect on lung volume mechanics should therefore be interpreted cautiously. These findings are consistent with current pathophysiological understanding, whereby ventilatory support primarily unloads respiratory muscles and enhances ventilatory efficiency without substantially modifying irreversible airflow limitation. The non-significant direction of FVC change without significant changes in FEV1 suggests that any mechanical benefit, if present, is more likely related to lung-volume mechanics than airway conductance. This is consistent with the physiological mechanism whereby mechanical unloading of the respiratory muscles reduces dynamic hyperinflation, thereby increasing the functional inspiratory capacity. As highlighted in recent studies on respiratory mechanics, the mitigation of hyperinflation is a key driver for improving exercise tolerance and reducing dyspnea, even in the presence of irreversible airflow limitation (13, 22). In terms of functional and symptom-related outcomes, the pooled analysis showed a significant improvement in exercise capacity, as measured by the 6-min walking distance (6MWD). This finding implies that improvements in gas exchange and lung volume mechanics may translate into measurable functional benefits (9, 15). Patient-reported outcomes suggested benefit in the available data: the St. George’s Respiratory Questionnaire-related index (SRI) improved significantly, and CAT scores were significantly reduced. Nevertheless, these findings should be interpreted cautiously because these instruments capture different dimensions of patient experience and symptom burden, and CAT data were available from a limited number of studies (14). Differences in what SRI and CAT measure may be attributed to the differing sensitivity of these instruments to the effects of ventilatory support. The SRI was specifically developed to assess health-related quality of life in patients with chronic respiratory failure receiving mechanical ventilation, making it more sensitive to improvements in sleep quality and respiratory muscle rest associated with nocturnal NIPPV (23). In contrast, the CAT is a more general measure of COPD symptom burden; the observed CAT improvement should therefore be interpreted as a broad symptom signal rather than a ventilation-specific quality-of-life effect. Compared with earlier meta-analyses, including the work by Struik et al. (11), which reported no significant long-term effects of NIPPV on gas exchange, lung function, or exercise capacity, the present analysis provides updated evidence supporting the physiological and functional benefits of nocturnal NIPPV. Differences in findings may be attributed to variations in study populations, ventilation strategies, outcome definitions, and, importantly, the timing of ventilatory support. Unlike previous reviews that included mixed daytime and nocturnal NIPPV use, the present study focused exclusively on nocturnal application, thereby reducing potential confounding related to treatment duration and daily activity. Substantial heterogeneity was observed across several outcomes, particularly blood gas parameters. This heterogeneity may be partly explained by differences in follow-up duration, baseline disease severity, ventilatory settings, and study design. Notably, the duration of follow-up varied considerably among the included studies, ranging from several weeks to 12 months or longer. Improvements in blood gas parameters and exercise capacity may be detectable over relatively short follow-up periods, whereas changes in airflow limitation and patient-reported symptom outcomes may require longer-term observation to become evident (19). The variability in follow-up duration should therefore be considered when interpreting both the magnitude and sustainability of treatment effects.

Because several outcomes showed substantial heterogeneity, particularly PaO2, arterial pH, FEV1% predicted, FEV1/FVC, and FVC, the pooled estimates should be interpreted as average effects across clinically diverse trials rather than as uniform treatment effects.

### Limitations

Several limitations of this meta-analysis should be acknowledged. First, the number of included studies was relatively limited (*n* = 8), which may reduce the precision of effect estimates and preclude a robust assessment of publication bias via funnel plots for certain outcomes. Second, the methodological quality of several included studies was rated as low to moderate, primarily due to insufficient reporting of random sequence generation or allocation concealment, indicating potential risks of bias. Third, although subgroup analyses based on ventilatory parameters—such as inspiratory and expiratory positive airway pressures—were intended, inconsistent reporting and insufficient data across the primary studies prevented a reliable exploration of these sources of heterogeneity. Finally, while our focus on nocturnal application reduced confounding variables related to treatment duration, it limits the generalizability of the findings to patients requiring daytime or continuous NIPPV support.

Additional methodological limitations should also be considered. The 2015 publication-year criterion was added after the initial PROSPERO registration and has now been documented as an eligibility amendment in the updated registration record. This amendment was intended to focus the synthesis on contemporary nocturnal home NIPPV practice after the shift toward PaCO2-targeted/high-intensity ventilation and current COPD background care; however, it may have excluded informative earlier trials and may reduce the generalizability of the review. Trial registries and grey-literature sources were not searched, certainty of evidence was not formally rated using GRADE, and leave-one-out sensitivity analyses, prespecified subgroup analyses, and mortality pooling could not be performed because of limited or non-comparable study-level data. These limitations may affect the reliability and applicability of the pooled estimates.

Additional limitations relate to external validity and clinical characterization. Only eight RCTs were included, six were conducted in China, and long-term oxygen therapy use, exacerbation history, GOLD stage, ventilatory settings, and ventilation targets were incompletely reported. These factors may limit generalizability and prevented reliable subgroup analyses by disease severity, oxygen therapy, or ventilatory strategy.

## Conclusion

Despite the aforementioned limitations, the present meta-analysis provides updated but cautious evidence regarding the therapeutic role of nocturnal NIPPV in stable COPD. While nocturnal NIPPV does not appear to substantially reverse fixed airflow limitation and the FVC effect was not statistically significant, it offers clinically meaningful benefits in correcting chronic hypercapnia, improving oxygenation, improving CAT and SRI scores in the available studies, and enhancing functional exercise capacity. These findings suggest that the primary value of nocturnal NIPPV may lie in physiological stabilization and functional preservation rather than the modification of underlying airway obstruction. Future high-quality randomized controlled trials characterized by standardized titration protocols, extended follow-up durations, and multidimensional patient-reported outcome measures are warranted. Such studies will be essential to refine patient selection criteria and optimize the personalized clinical application of nocturnal NIPPV in long-term COPD management.

## Data Availability

The original contributions presented in the study are included in the article/Supplementary material, further inquiries can be directed to the corresponding author/s.

## References

[1] Agustí A Celli BR Criner GJ Halpin D Anzueto A Barnes P . Global initiative for chronic obstructive lung disease 2023 report: GOLD executive summary. Eur Respir J. (2023) 61:2300239. doi: 10.1183/13993003.00239-2023, 36858443 PMC10066569

[2] Csoma B Vulpi MR Dragonieri S Bentley A Felton T Lázár Z . Hypercapnia in COPD: causes, consequences, and therapy. J Clin Med. (2022) 11:3180. doi: 10.3390/jcm11113180, 35683563 PMC9181664

[3] Osadnik CR Tee VS Carson-Chahhoud KV Picot J Wedzicha JA Smith BJ . Non-invasive ventilation for the management of acute hypercapnic respiratory failure due to exacerbation of chronic obstructive pulmonary disease. Cochrane Database Syst Rev. (2017) 2017:Cd004104. doi: 10.1002/14651858.CD004104.pub4, 28702957 PMC6483555

[4] Farmer MJS Callahan CD Hughes AM Riska KL Hill NS. Applying noninvasive ventilation in treatment of acute exacerbation of COPD using evidence-based Interprofessional clinical practice. Chest. (2024) 165:1469–80. doi: 10.1016/j.chest.2024.02.040, 38417700 PMC11177098

[5] Ergan B Oczkowski S Rochwerg B Carlucci A Chatwin M Clini E . European Respiratory Society guidelines on long-term home non-invasive ventilation for management of COPD. Eur Respir J. (2019) 54:1901003. doi: 10.1183/13993003.01003-2019, 31467119

[6] Orr JE Coleman JM III McSparron JI Owens RL Macrea M Drummond MB . Summary for clinicians: clinical practice guideline for long-term noninvasive ventilation in chronic stable Hypercapnic chronic obstructive pulmonary disease. Ann Am Thorac Soc. (2021) 18:395–8. doi: 10.1513/AnnalsATS.202009-1171AG, 33326340

[7] Macrea M Oczkowski S Rochwerg B Branson RD Celli B Coleman JM III . Long-term noninvasive ventilation in chronic stable Hypercapnic chronic obstructive pulmonary disease. An official American Thoracic Society clinical practice guideline. Am J Respir Crit Care Med. (2020) 202:e74–87. doi: 10.1164/rccm.202006-2382ST, 32795139 PMC7427384

[8] Wu Z Luo Z Luo Z Ge J Jin J Cao Z . Baseline level and reduction in PaCO2 are associated with the treatment effect of long-term home noninvasive positive pressure ventilation in stable Hypercapnic patients with COPD: a systematic review and Meta-analysis of randomized controlled trials. Int J Chron Obstruct Pulmon Dis. (2022) 17:719–33. doi: 10.2147/copd.S344962, 35418751 PMC8995153

[9] Xie S Li X Liu Y Huang J Yang F. Effect of home noninvasive positive pressure ventilation combined with pulmonary rehabilitation on dyspnea severity and quality of life in patients with severe stable chronic obstructive pulmonary disease combined with chronic type II respiratory failure: a randomized controlled trial. BMC Pulm Med. (2025) 25:185. doi: 10.1186/s12890-025-03656-3, 40259286 PMC12013138

[10] Park SY Yoo KH Park YB Rhee CK Park J Park HY . The long-term efficacy of domiciliary noninvasive positive-pressure ventilation in chronic obstructive pulmonary disease: a Meta-analysis of randomized controlled trials. Tuberc Respir Dis (Seoul). (2022) 85:47–55. doi: 10.4046/trd.2021.0062, 34775737 PMC8743632

[11] Struik FM Lacasse Y Goldstein R Kerstjens HAM Wijkstra PJ Cochrane Airways Group. Nocturnal non-invasive positive pressure ventilation for stable chronic obstructive pulmonary disease. Cochrane Database Syst Rev. (2013) 2013:Cd002878. doi: 10.1002/14651858.CD002878.pub2, 23766138 PMC6999800

[12] Sterne JAC Savović J Page MJ Elbers RG Blencowe NS Boutron I . RoB 2: a revised tool for assessing risk of bias in randomised trials. BMJ. (2019) 366:l4898. doi: 10.1136/bmj.l4898, 31462531

[13] Duiverman ML Vonk JM Bladder G van Melle JP Nieuwenhuis J Hazenberg A . Home initiation of chronic non-invasive ventilation in COPD patients with chronic hypercapnic respiratory failure: a randomised controlled trial. Thorax. (2020) 75:244–52. doi: 10.1136/thoraxjnl-2019-213303, 31484786 PMC7063397

[14] Zhou LQ Li XY Li Y Guo BP Guan LL Chen X . Inspiratory muscle training followed by non-invasive positive pressure ventilation in patients with severe chronic obstructive pulmonary disease: a randomized controlled trial. J South Med Univ. (2016) 36:1069–74. 27578574

[15] Zhou YJ Miao XH Zhou Y Zhuang ZF Xu J. Effect of non-invasive positive pressure ventilation at daytime or nighttime on pulmonary rehabilitation in patients with stable chronic obstructive pulmonary disease. Pract Geriatr. (2019) 33:269–72. doi: 10.3969/j.issn.1003-9198.2019.03.016

[16] Xu P. Analysis of the effect of nocturnal non-invasive positive pressure ventilation in the treatment of patients with chronic obstructive pulmonary disease combined with hypercapnic respiratory failure. World J Complex Med. (2020) 6:102–5. doi: 10.11966/j.issn.2095-994X.2020.06.01.33

[17] Cheng N Peng S. Efficacy of nocturnal non-invasive positive pressure ventilation in the treatment of chronic obstructive pulmonary disease patients with hypercapnic respiratory failure. Chin J Gerontol. (2018) 38:1117–9. doi: 10.3969/j.issn.1005-9202.2018.05.036

[18] Ge NH Cai XZ Xiao CC. Analysis of the impact of non-invasive ventilation on the quality of life in patients with COPD combined with pulmonary hypertension. China Med Pharm. (2017) 7:248–51.

[19] Murphy PB Rehal S Arbane G Bourke S Calverley PMA Crook AM . Effect of home noninvasive ventilation with oxygen therapy vs oxygen therapy alone on hospital readmission or death after an acute COPD exacerbation: a randomized clinical trial. JAMA. (2017) 317:2177–86. doi: 10.1001/jama.2017.4451, 28528348 PMC5710342

[20] Sharma M Joshi S Banjade P Ghamande SA Surani S. Global initiative for chronic obstructive lung disease (GOLD) 2023 guidelines reviewed. Open Respir Med J. (2024) 18:e18743064279064. doi: 10.2174/0118743064279064231227070344, 38660684 PMC11037508

[21] Köhnlein T Windisch W Köhler D Drabik A Geiseler J Hartl S . Non-invasive positive pressure ventilation for the treatment of severe stable chronic obstructive pulmonary disease: a prospective, multicentre, randomised, controlled clinical trial. Lancet Respir Med. (2014) 2:698–705. doi: 10.1016/s2213-2600(14)70153-5, 25066329

[22] O'Donnell DE James MD Milne KM Neder JA. The pathophysiology of dyspnea and exercise intolerance in chronic obstructive pulmonary disease. Clin Chest Med. (2019) 40:343–66. doi: 10.1016/j.ccm.2019.02.007, 31078214

[23] Windisch W Freidel K Schucher B Baumann H Wiebel M Matthys H . The severe respiratory insufficiency (SRI) questionnaire: a specific measure of health-related quality of life in patients receiving home mechanical ventilation. J Clin Epidemiol. (2003) 56:752–9. doi: 10.1016/s0895-4356(03)00088-x, 12954467

