# Supplementary material for: The safety and efficacy of nocturnal noninvasive positive pressure ventilation in patients with chronic obstructive pulmonary disease: a systematic review and meta-analysis
Source: Front Med (Lausanne). 2026 Jul 14;13:1882152. doi: 10.3389/fmed.2026.1882152 (PMC13407778; doi:10.3389/fmed.2026.1882152)
Supplement: Supplementary file 1 [file Table_1.doc]

**eTable1.** The complete search strategy.

| Database | Detailed Search Strategy | Date | Results |
| --- | --- | --- | --- |
| Pubmed | ("Noninvasive Ventilation"[MeSH Terms] OR ("noninvasive ventilations"[Title/Abstract] OR "ventilation noninvasive"[Title/Abstract] OR (("ventilated"[All Fields] OR "ventilates"[All Fields] OR "ventilating"[All Fields] OR "Ventilation"[MeSH Terms] OR "Ventilation"[All Fields] OR "ventilate"[All Fields] OR "Ventilations"[All Fields] OR "ventilator s"[All Fields] OR "ventilators, mechanical"[MeSH Terms] OR ("ventilators"[All Fields] AND "mechanical"[All Fields]) OR "mechanical ventilators"[All Fields] OR "ventilator"[All Fields] OR "ventilators"[All Fields] OR "ventillation"[All Fields]) AND "Noninvasive"[Title/Abstract]) OR "non invasive ventilation"[Title/Abstract] OR "non invasive ventilations"[Title/Abstract] OR "ventilation non invasive"[Title/Abstract] OR (("ventilated"[All Fields] OR "ventilates"[All Fields] OR "ventilating"[All Fields] OR "Ventilation"[MeSH Terms] OR "Ventilation"[All Fields] OR "ventilate"[All Fields] OR "Ventilations"[All Fields] OR "ventilator s"[All Fields] OR "ventilators, mechanical"[MeSH Terms] OR ("ventilators"[All Fields] AND "mechanical"[All Fields]) OR "mechanical ventilators"[All Fields] OR "ventilator"[All Fields] OR "ventilators"[All Fields] OR "ventillation"[All Fields]) AND "Non-Invasive"[Title/Abstract]) OR "non invasive ventilation"[Title/Abstract] OR "non invasive ventilations"[Title/Abstract] OR "ventilation non invasive"[Title/Abstract] OR (("ventilated"[All Fields] OR "ventilates"[All Fields] OR "ventilating"[All Fields] OR "Ventilation"[MeSH Terms] OR "Ventilation"[All Fields] OR "ventilate"[All Fields] OR "Ventilations"[All Fields] OR "ventilator s"[All Fields] OR "ventilators, mechanical"[MeSH Terms] OR ("ventilators"[All Fields] AND "mechanical"[All Fields]) OR "mechanical ventilators"[All Fields] OR "ventilator"[All Fields] OR "ventilators"[All Fields] OR "ventillation"[All Fields]) AND "Non-Invasive"[Title/Abstract]))) AND ("pulmonary disease, chronic obstructive"[MeSH Terms] OR ("chronic obstructive pulmonary diseases"[Title/Abstract] OR "COPD"[Title/Abstract] OR "chronic obstructive lung disease"[Title/Abstract] OR "chronic obstructive pulmonary disease"[Title/Abstract] OR "COAD"[Title/Abstract] OR "chronic obstructive airway disease"[Title/Abstract] OR "airflow obstruction chronic"[Title/Abstract] OR "airflow obstructions chronic"[Title/Abstract] OR "chronic airflow obstructions"[Title/Abstract] OR "chronic airflow obstruction"[Title/Abstract])) AND ("randomized controlled trial"[Publication Type] OR "randomized"[Title/Abstract] OR "placebo"[Title/Abstract]) | July 26, 2025 | 503 |
| Cochrane Library | (Noninvasive Ventilation):ti,ab,kw AND (Pulmonary Disease, Chronic Obstructive):ti,ab,kw AND (nocturnal):ti,ab,kw | July 26 2025 | 83 |
| Embase | ('noninvasive ventilation'/exp OR (noninvasive AND ventilation:ab,ti)) AND ('chronic obstructive lung disease'/exp OR (chronic AND obstructive AND lung AND disease:ab,ti)) AND 'clinical trial'/exp AND nocturnal:ab,ti | July 26, 2025 | 65 |
| Web of Science | TS=(("noninvasive ventilation" OR "non-invasive ventilation" OR NIPPV OR NIV) AND (COPD OR "chronic obstructive pulmonary disease" OR "chronic obstructive lung disease") AND (nocturnal OR night* OR sleep*) AND (random* OR trial OR placebo)) | July 26 2025 | 70 |
| CNKI | （主题：无创通气 + 无创正压通气）AND（主题：慢性阻塞性肺疾病）AND（主题：夜间） | July 26 2025 | 50 |
| Wanfang | 主题:(无创通气 OR 无创正压通气) and 主题:(慢性阻塞性肺疾病) and 主题:(夜间) | July 26, 2025 | 77 |

**eTable 2.** Pre-2015 RCTs excluded at full-text screening solely on the basis of publication year (n = 2).

| **Study** | **Journal, Year** | **Sample Size** | **IPAP / EPAP (cmH₂O)** | **Population** | **Main Findings** | **Rationale for Exclusion** |
| --- | --- | --- | --- | --- | --- | --- |
| McEvoy et al. | Thorax 2009; 64(7): 561–566 | 144 (72 vs 72) | 13 / 5 | Stable hypercapnic COPD (FEV₁ <1.5 L or <50% pred; PaCO₂ >46 mmHg); on LTOT ≥3 months | Marginal survival benefit (adjusted HR 0.63, 95% CI 0.40–0.99, p = 0.045); worsened QoL; mean FU 2.21 yr | Published before 2015.  Low-intensity IPAP (13 cmH₂O) without PaCO₂-targeted titration; pre-GOLD 2017 background care. |
| Clini et al. | Eur Respir J 2002; 20(3): 529–538 | 90 (randomized from 122) | 14 / 2 | Stable COPD with chronic hypercapnia; LTOT ≥6 months; 20 centres, Italy | Reduced hospital admissions (−45%) and ICU admissions (−75%); no survival benefit; 2-yr FU | Published before 2015.  Low-intensity IPAP (14 cmH₂O) without PaCO₂-targeted titration; pre-modern COPD pharmacotherapy. |

**Abbreviations:** IPAP, inspiratory positive airway pressure; EPAP, expiratory positive airway pressure; LTOT, long-term oxygen therapy; FU, follow-up; HR, hazard ratio; CI, confidence interval; QoL, quality of life; ICU, intensive care unit; FEV₁, forced expiratory volume in 1 second; PaCO₂, partial pressure of arterial carbon dioxide.


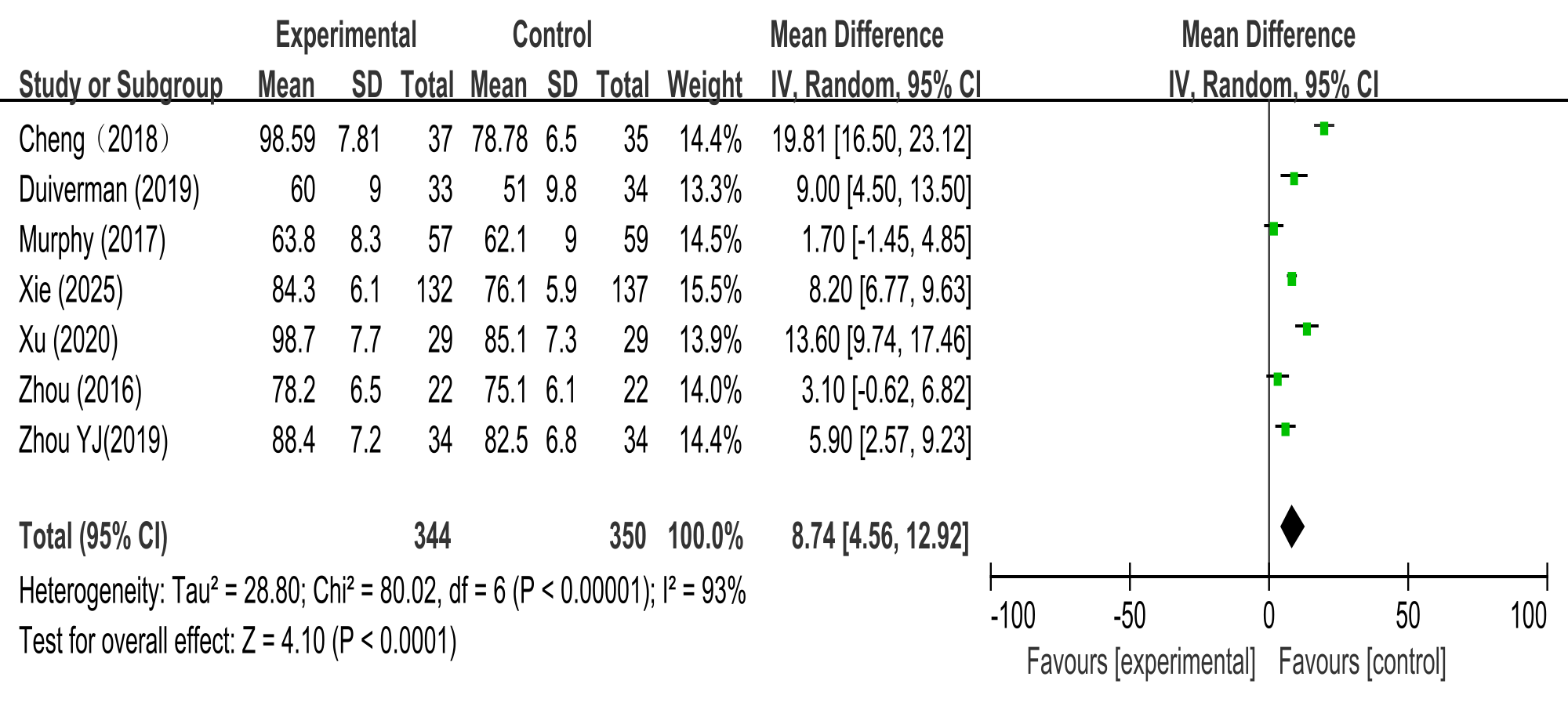


Supplementary Fig. 1. Forest plotPaO2.Description: This plot illustrates the effect of nocturnal NIPPV on arterial oxygen tension (PaO_2_).Statistical Analysis: A random-effects model was utilized given the very high heterogeneity observed among the seven included studies (*I^2^* = 93%, P < 0.00001).Results: The pooled analysis showed a statistically significant improvement in PaO_2_ for the NIPPV group (MD = 8.74, 95% CI: 4.56 to 12.92; *P* < 0.0001).


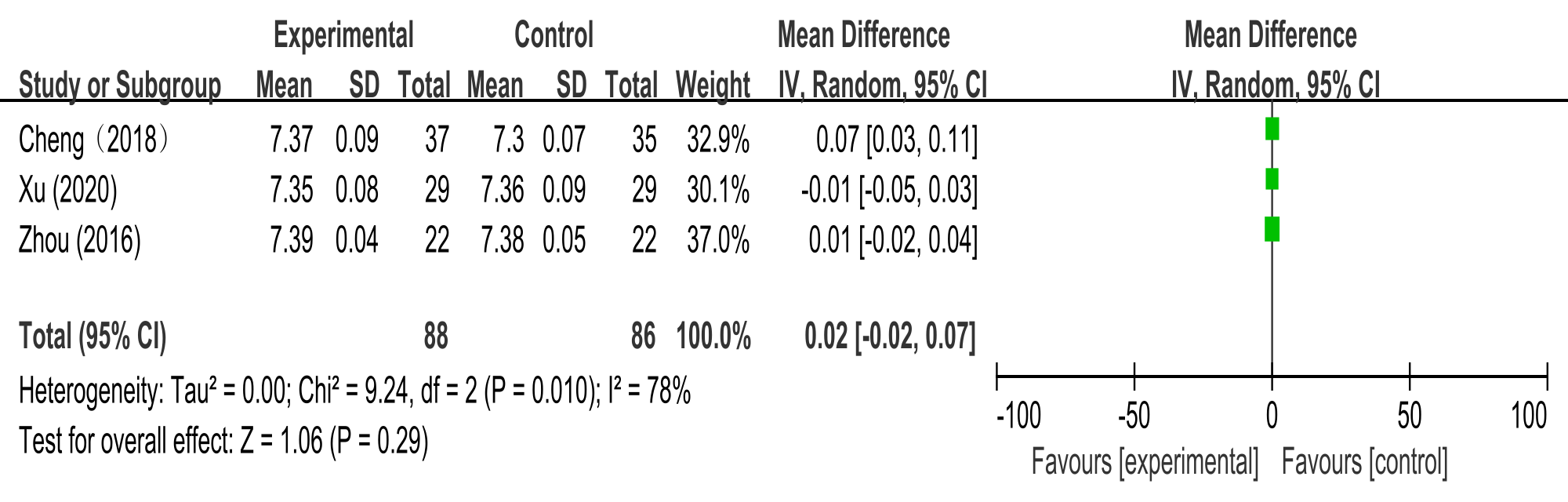


Supplementary Fig. 2. Forest plot of arterial pH.Comparison of arterial pH between the nocturnal NIPPV group and the conventional therapy group. A random-effects model was applied due to significant heterogeneity observed among the included studies (*I^2^* = 78%, *P* = 0.01). The pooled result (MD = 0.02, 95% CI: -0.02 to 0.07; *P* = 0.29) indicates no statistically significant difference in arterial pH between the two groups.


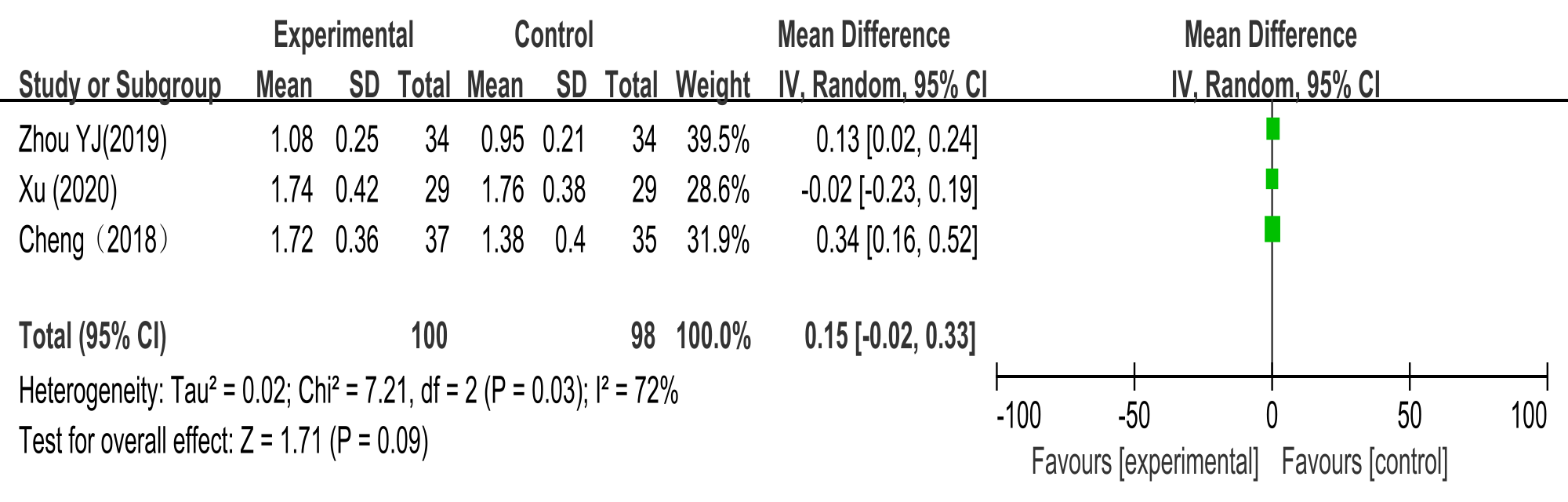


Supplementary Fig. 3. Forest plot of FVC (L). Description: This forest plot evaluates the impact of nocturnal NIPPV on forced vital capacity (FVC). Statistical analysis: A random-effects model was applied due to substantial statistical heterogeneity among the included studies (I^2^ = 72%, *P* = 0.03). Results: The pooled mean difference (MD) was 0.15 (95% CI -0.02 to 0.33; *P* = 0.09), indicating that nocturnal NIPPV did not lead to a statistically significant improvement in FVC compared with conventional therapy.


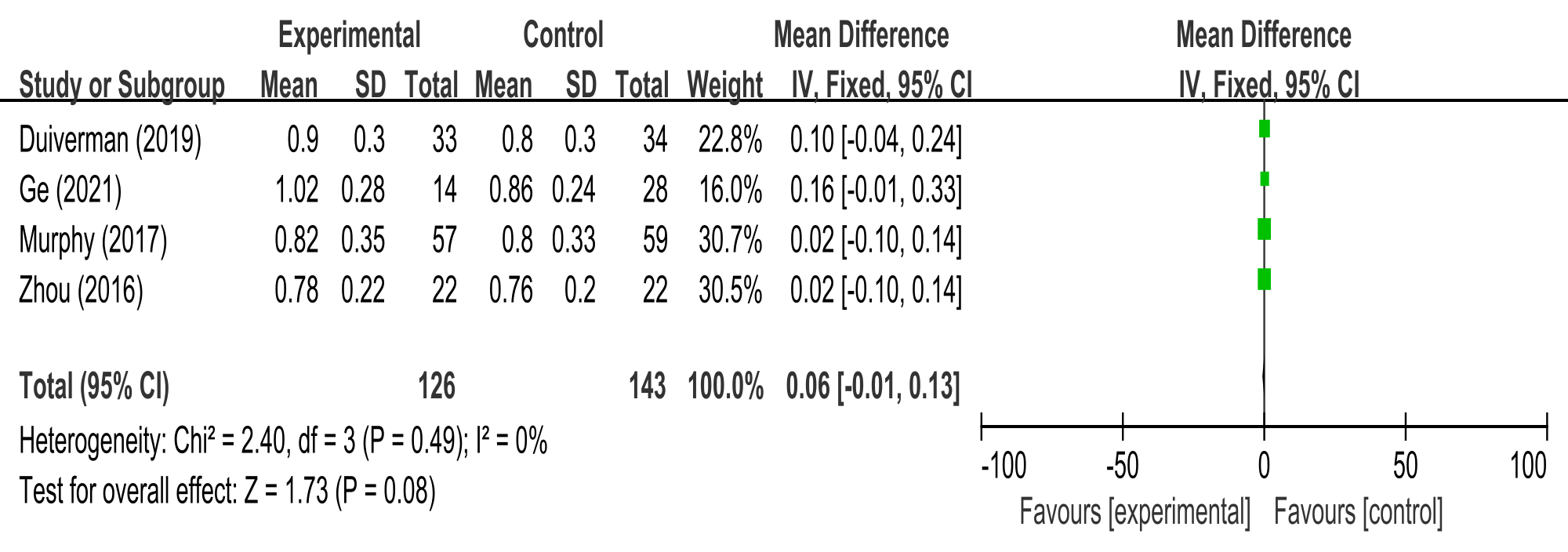


Supplementary Fig. 4. Forest plot FEV1(L).Description: This forest plot compares the forced expiratory volume in one second (FEV ) measured in liters between the nocturnal NIPPV group and the conventional therapy group.Statistical Analysis: A fixed-effects model was applied as no statistical heterogeneity was observed among the included studies (*I^2^* =0%, P=0.49).Results: The pooled mean difference (MD) was 0.06 (95% CI: -0.01 to 0.13; *P*=0.08), indicating no statistically significant difference in FEV1 (L) between the two groups.


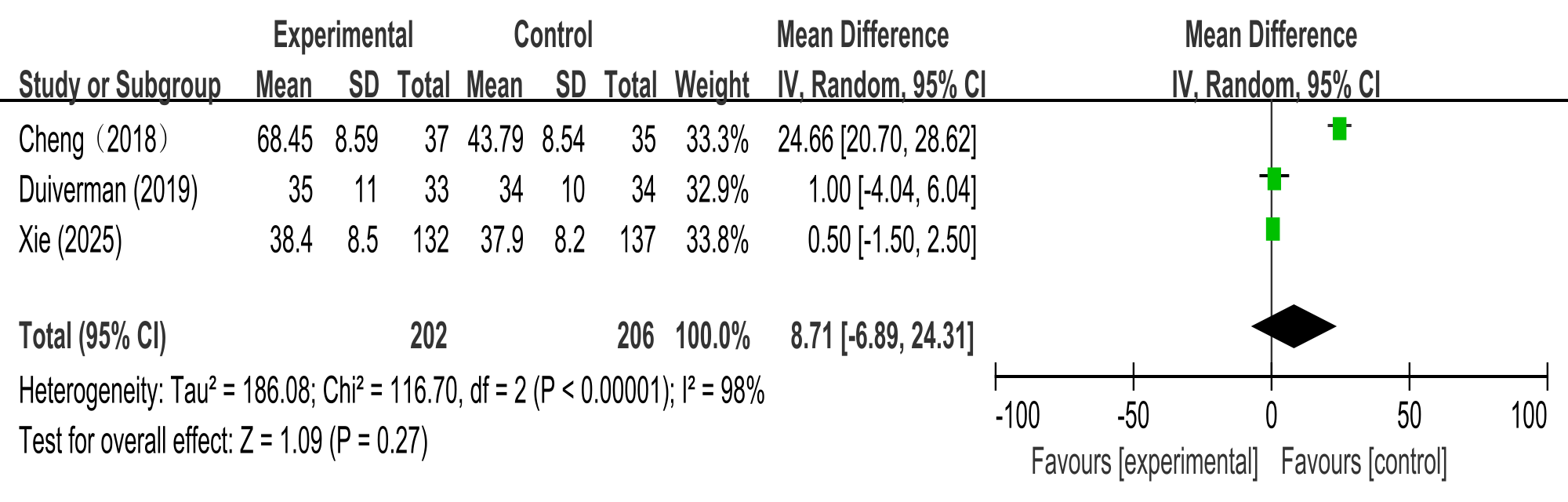


Supplementary Fig. 5. Forest plot FEV1（%）.Description: This forest plot evaluates the impact of nocturnal NIPPV on the forced expiratory volume in one second (FEV1) expressed as a percentage of predicted values. Statistical Analysis: A random-effects model was applied due to extreme statistical heterogeneity among the included studies (*I^2^* = 98%, *P* < 0.00001). Results: The pooled mean difference (MD) was 8.71 (95% CI: -6.89 to 24.31; *P* = 0.27), indicating that nocturnal NIPPV did not lead to a statistically significant improvement in FEV1 (% predicted) compared to conventional therapy.


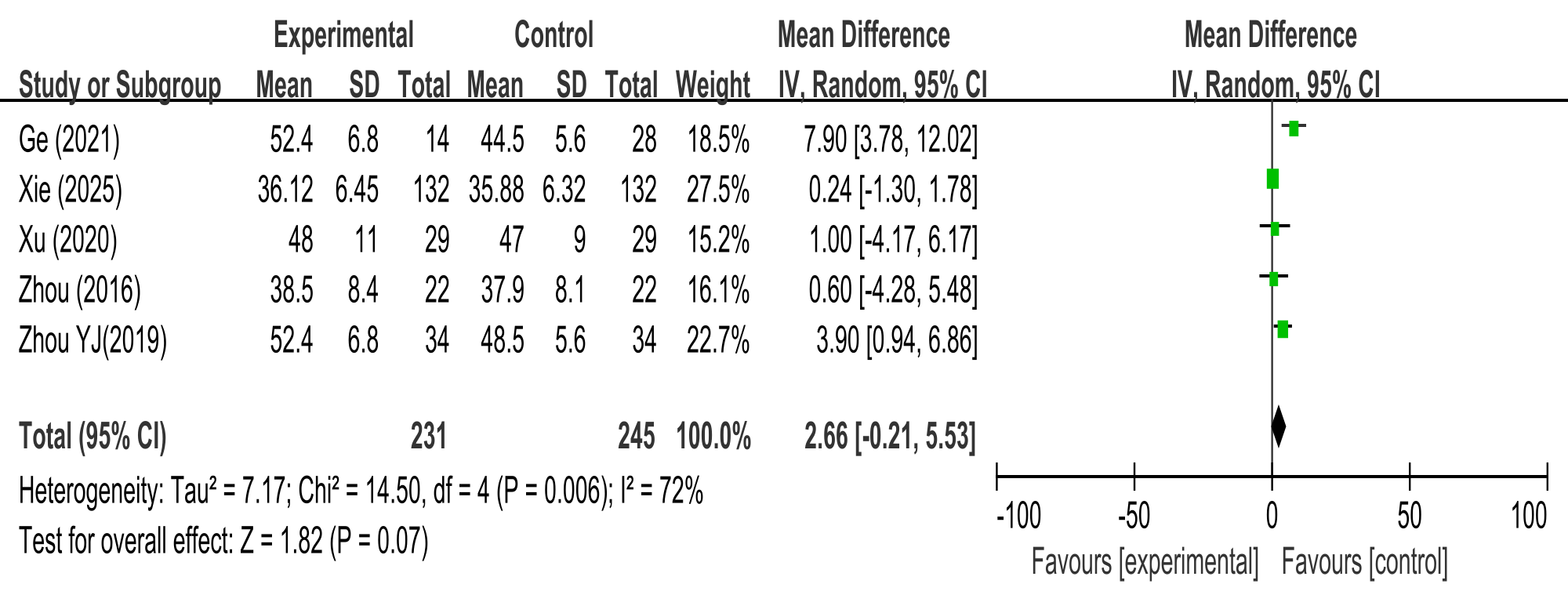


Supplementary Fig. 6. Forest plot FEV1/FVC（%）.Description: This forest plot evaluates the impact of nocturnal NIPPV on the ratio of forced expiratory volume in one second to forced vital capacity (FEV1/FVC).Statistical Analysis: A random-effects model was applied due to substantial statistical heterogeneity observed among the five included studies (*I^2^* = 72%, *P* = 0.006).Results: The pooled mean difference (MD) was 2.66 (95% CI: -0.21 to 5.53; *P* = 0.07), indicating that nocturnal NIPPV did not lead to a statistically significant improvement in the FEV1/FVC ratio compared to conventional therapy.


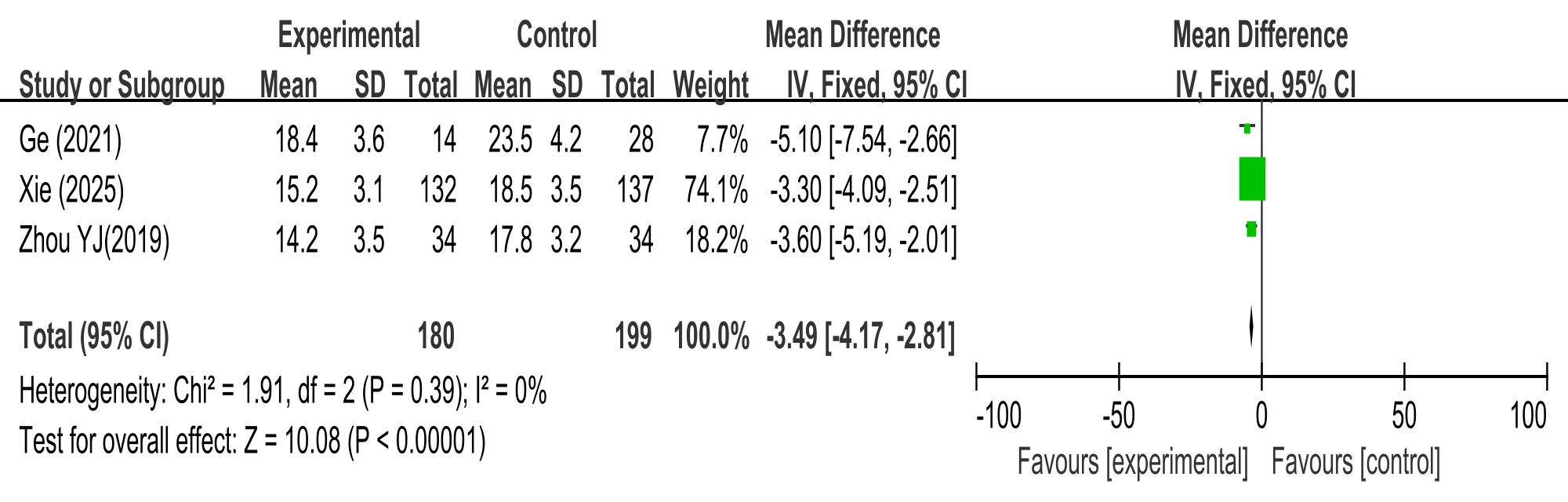


Supplementary Fig. 7. Forest plot of CAT (points). Description: This forest plot evaluates the impact of nocturnal NIPPV on patient-reported symptom burden using CAT scores. Statistical analysis: A fixed-effects model was applied, as no statistical heterogeneity was observed among the included studies (I^2^ = 0%, *P* = 0.39). Results: The pooled analysis demonstrated a statistically significant reduction in CAT scores in the nocturnal NIPPV group compared with the conventional therapy group (MD = -3.49, 95% CI -4.17 to -2.81; *P* < 0.00001).
